# Glycogen Synthase Kinase-3β (GSK3β): Role in the Mechanism of Action of Lithium and Potential as a Predictive Biomarker of Therapeutic Response in Bipolar Disorder

**DOI:** 10.1192/j.eurpsy.2026.11860

**Published:** 2026-07-31

**Authors:** I. Mosbah, H. Ghabi, S. Abdelmoumen, H. Nefzi, R. kammoun, M. Karoui, F. Ellouze

**Affiliations:** 1 Department of Psychiatry G, Razi hospital, Manouba; 2 El Manar University, Faculty of Medicine of Tunis, tunis, Tunisia

## Abstract

**Introduction:**

Bipolar disorder is a chronic psychiatric condition characterized by alternating depressive, manic, or mixed episodes. Lithium remains a gold-standard treatment, effective both in managing acute episodes and in preventing relapses, while also reducing suicide risk. However, substantial interindividual variability in lithium response poses a major clinical challenge, with only about one-third of patients achieving optimal outcomes. Identifying predictive biomarkers is therefore essential to guide more personalized treatment strategies.

**Objectives:**

This systematic review aimed to explore the role of glycogen synthase kinase-3 beta (GSK3β) in the mechanism of action of lithium and to assess its potential as a predictive biomarker of therapeutic response in bipolar disorder.

**Methods:**

Following PRISMA guidelines, a systematic search was conducted in PubMed, ScienceDirect, Embase, and Google Scholar (1999–2025) using keywords related to lithium, GSK3β, and bipolar disorder. Eligible studies included adult patients treated with lithium and reporting data on GSK3β activity, phosphorylation, or genetic polymorphisms in relation to clinical response.

**Results:**

Experimental studies confirm that lithium directly inhibits GSK3β, either by competing with magnesium or via the PI3K/Akt pathway, leading to serine-9 phosphorylation and neuroprotective effects. Clinically, increased levels of phosphorylated GSK3β have been correlated with symptomatic improvement. In addition, several GSK3β gene polymorphisms, particularly −50T/C (rs334558), have been linked to differences in lithium response, with the C allele associated with greater long-term efficacy.

**Conclusions:**

GSK3β emerges as a central mediator of lithium’s mood-stabilizing effects and a promising biomarker of therapeutic response. Integrating functional and genetic markers could ultimately allow for patient stratification and foster a more personalized approach to the treatment of bipolar disorder.

**Disclosure of Interest:**

None Declared

